# Drug-Induced Sleep Endoscopy in Pediatric Obstructive Sleep Apnea: Clinical Applications, Classification Systems, and Anesthetic Considerations

**DOI:** 10.3390/jcm15176630

**Published:** 2026-08-27

**Authors:** Patryk Oskar Manycz, Monika Morawska-Kochman, Jakub Zieliński

**Affiliations:** Department of Otolaryngology, Wroclaw Medical University, Pasteura 1, 50-369 Wrocław, Poland

**Keywords:** drug-induced sleep endoscopy, obstructive sleep apnea, children, upper airway, sedation

## Abstract

**Background/Objectives:** Drug-induced sleep endoscopy (DISE) enables dynamic, real-time visualization of the level, severity, and configuration of upper airway collapse during pharmacologically induced sleep, providing anatomical information that polysomnography cannot. It is increasingly used to guide individualized surgical planning in obstructive sleep apnea (OSA), particularly in children with persistent OSA after adenotonsillectomy or at elevated risk of surgical failure. However, the absence of a universally accepted pediatric-specific classification system and standardized protocol continues to limit comparability across studies and the development of evidence-based treatment algorithms. This review evaluates the role of DISE in pediatric OSA, focusing on airway assessment, classification systems, anesthesia protocols, and its impact on surgical decision-making. **Methods:** A PubMed literature review (2013–2025) identified 42 pediatric-specific studies, including cohort studies, systematic reviews, and meta-analyses. Data were extracted regarding indications, classification scales, anesthetic techniques, safety, and surgical outcomes. **Results:** DISE provides dynamic visualization of multilevel obstructions often missed during awake examinations. While adult-derived systems such as VOTE are used, pediatric-specific tools (e.g., Chan–Parikh, NAVOTEL, PedDISE-8, IPSES) offer more age-appropriate assessments. Anesthetic choice is a key factor; dexmedetomidine, alone or with ketamine, best approximates non-rapid eye movement (NREM) sleep while maintaining airway tone and stability. DISE findings altered surgical plans in 30–60% of patients, facilitating targeted procedures such as supraglottoplasty, lingual tonsillectomy, and epiglottopexy. **Conclusions:** DISE is a valuable diagnostic and decision-support tool for complex pediatric OSA. Clinical utility depends on standardized assessment and anesthesia. Further multicenter studies are required to validate classification systems and determine long-term impacts on quality of life.

## 1. Introduction

Obstructive sleep apnea (OSA) is a common disorder characterized by recurrent episodes of partial or complete upper airway obstruction during sleep, resulting in intermittent hypoxia, sleep fragmentation, and disrupted ventilation [1,2,3,4,5]. Polysomnography (PSG) remains the gold standard for diagnosing OSA. In pediatric patients, OSA is typically diagnosed when the apnea–hypopnea index (AHI) is ≥1 event per hour on overnight PSG [3,6,7,8]. While PSG provides a comprehensive physiological assessment of sleep-disordered breathing, it does not allow for precise identification of the anatomical level or mechanism of upper airway obstruction [6]. Drug-induced sleep endoscopy (DISE) was first introduced by Croft and Pringle in 1991 as a diagnostic method for dynamic evaluation of the upper airway during pharmacologically induced sleep [1]. The procedure involves flexible endoscopic visualization of the airway while the patient is under sedation that approximates natural sleep, most commonly using agents such as propofol or dexmedetomidine [9,10,11,12,13,14]. DISE enables real-time assessment of the level, severity, and configuration of airway collapse, providing information that is highly valuable for individualized surgical planning [6,10,13,15,16]. This is particularly relevant in patients who are unable to tolerate continuous positive airway pressure (CPAP) therapy or in whom CPAP has proven ineffective [17]. To facilitate standardized interpretation and comparison of DISE findings, several classification systems have been developed [3,11,18]. The most widely used system worldwide is the VOTE classification, which evaluates obstruction at four anatomical levels: the velum, oropharynx, tongue base, and epiglottis [2,11,16,19,20]. The VOTE system grades both the degree of airway collapse (0 = none, 1 = partial, 2 = complete) and the pattern of collapse (anteroposterior, lateral, or concentric). Although DISE is currently regarded as the most informative technique for dynamic assessment of upper airway obstruction during sleep [3], there is no universally accepted classification system or standardized methodology for performing and interpreting the examination [14,21,22]. Interpretation of DISE findings is largely based on the examiner’s subjective assessment, which may result in interobserver variability and inconsistencies in surgical decision-making and outcome prediction [3,14,21]. Consequently, despite its recognized diagnostic value, the role of DISE in reliably predicting treatment outcomes remains an area of ongoing investigation [14,23]. In the pediatric population, DISE has emerged as a particularly valuable diagnostic tool for evaluating upper airway obstruction in children with OSA [9,24]. Its use is especially relevant in children with persistent OSA following prior surgical interventions, such as adenoidectomy or adenotonsillectomy (AT), as well as in patients at increased risk of AT failure, including those with small palatine tonsils, Down syndrome, obesity, or craniofacial anomalies [11,20,24]. Despite its increasing clinical application, there is currently no universally accepted pediatric-specific classification system or standardized assessment protocol for DISE [25,26]. This lack of uniformity limits comparability between studies and hinders the development of evidence-based treatment algorithms for children with OSA. The present review aims to synthesize current evidence regarding the application of DISE in the pediatric population, with a particular focus on indications for use, airway classification systems, anesthetic protocols, and the impact of DISE findings on surgical management and outcomes.

## 2. Materials and Methods

A structured literature review was conducted using a predefined search strategy, designed to identify and synthesize available evidence on the use of DISE in the pediatric population. The PubMed database was searched for relevant publications from 2013 to 2025, with particular emphasis on studies published between 2020 and 2025 to capture the most recent developments in the field. Only articles published in English were considered. The search strategy combined Medical Subject Headings (MeSH) terms and free-text keywords, including “drug-induced sleep endoscopy,” “DISE,” “pediatric,” “children,” “obstructive sleep apnea,” and “sleep-disordered breathing.” The reference lists of selected articles and relevant review papers were also screened to identify additional studies that may not have been captured during the initial database search. Study selection was guided by predefined inclusion and exclusion criteria. Eligible studies included pediatric patients aged 0–18 years diagnosed with obstructive sleep apnea or other forms of sleep-disordered breathing who underwent evaluation with DISE of the upper airway. Studies were required to report DISE findings alone or in comparison with awake physical examination, PSG performed before and/or after intervention, or between distinct clinical subgroups (e.g., obese versus non-obese children or children with Down syndrome). Eligible study designs included prospective and retrospective cohort studies, observational and case–control studies, systematic reviews, meta-analyses, and expert consensus statements. Outcomes of interest comprised anatomical sites and patterns of airway obstruction identified during DISE, changes in surgical treatment planning based on DISE findings, polysomnographic parameters (including AHI, obstructive AHI, and oxygen saturation), procedural safety, and anesthetic protocols used during the examination. Studies focusing exclusively on adult populations (≥18 years of age), publications lacking original clinical data, and articles not specifically addressing the DISE procedure (e.g., studies focused solely on CPAP therapy without endoscopic assessment) were excluded from the analysis. To ensure a comprehensive and transparent presentation of the synthesized evidence, the detailed characteristics of all included pediatric studies—including patient demographics, prior surgical history, anesthetic protocols, scoring systems, and primary clinical outcomes—are systematically outlined in Table 1.

## 3. Results

Direct comparisons between studies should be interpreted with caution due to varying definitions of surgical success; to provide clarity, comprehensive preoperative and postoperative results are tracked in the Table 1 for each included study.

### 3.1. Population Size, Age, and Sex Distribution

The included studies demonstrated substantial heterogeneity in study design, sample size, patient age, and sex distribution, reflecting the wide clinical spectrum of pediatric OSA. Across the included original clinical studies in this review, the cumulative number of pediatric patients exceeded 1400. Sample sizes ranged from small case series—such as pilot studies evaluating long-range optical coherence tomography in 2 patients [27] or CPAP titration during DISE in 12 patients [28]—to large retrospective cohorts including more than 100 patients [29,30,31,32]. In addition, systematic reviews and meta-analyses encompassed broader populations, reporting aggregated sample sizes ranging from 761 [4] to 996 [33] children. Patient age spanned the entire pediatric range, from infancy to late adolescence. The youngest cohorts included infants with a mean age of 9.6 months [9], whereas the oldest participants were up to 18 years of age [6]. In most studies, the mean or median age ranged from approximately 3.5 to 9 years [19,20,25,29,30,31,32,34,35,36,37]. With regard to sex distribution, male patients predominated in most cohorts, typically accounting for 55–69% of participants [19,29,30,31,32,36,37,38,39,40,41,42,43,44]. Only a small number of studies reported a female predominance, with girls comprising 52–75% of the study population [20,28,34,35].

### 3.2. Pediatric-Specific Classification Systems

Regardless of the system used, expert consensus emphasizes that DISE assessment should systematically document three key parameters at each anatomical site: the anatomical location of obstruction, the degree of obstruction (severity), and the pattern or configuration of collapse (e.g., anteroposterior, lateral, or concentric) [2,11]. Many classification systems, including recently developed tools, utilize a three-point Likert scale to assess obstruction severity based on the maximal observed degree of airway narrowing: <50%, 50–90%, and >90% narrowing [25,26]. An overview and comparison of the most frequently cited and validated DISE assessment systems used in the pediatric population are presented below, with a summary of the principal advantages and limitations of each system provided in Table 2.

#### 3.2.1. VOTE Classification

The VOTE classification (Velum, Oropharynx, Tongue base, Epiglottis) is the most widely used DISE assessment system worldwide and was originally developed for adult patients [11,21,51]. It evaluates four primary anatomical sites of obstruction and characterizes both the degree of collapse (0 = none, 1 = partial, 2 = complete) and the collapse configuration (anteroposterior, lateral, or concentric). In pediatric patients, this system has been criticized for being incomplete, as it omits anatomical sites that are particularly relevant to airway obstruction in children, including the nasal cavity, adenoids, and laryngeal or supraglottic structures. As a result, the VOTE classification may underestimate the complexity and extent of airway obstruction in the pediatric population [11,18,25,36].

#### 3.2.2. The Chan–Parikh System

The Chan–Parikh system is one of the earliest DISE classification systems developed specifically for pediatric patients and includes five anatomical sites: the adenoids, velum, lateral pharyngeal walls/palatine tonsils, tongue base, and supraglottis [21,45]. Unlike the VOTE classification, the Chan–Parikh system employs a four-point grading scale (0–3) to quantify the degree of obstruction [44]. Several studies have demonstrated that the total Chan–Parikh score correlates with OSA severity, as measured by obstructive AHI and minimum oxygen saturation. However, as with the VOTE classification, this system does not include an assessment of the nasal cavity [11,25].

#### 3.2.3. The Sleep Endoscopy Rating Scale (SERS)

The Sleep Endoscopy Rating Scale [46] was designed to provide a more comprehensive airway assessment by including six anatomical regions, including the nasal cavity. However, hypopharyngeal structures such as the tongue base and epiglottis are grouped into a single level (the hypopharynx), which may limit anatomical precision [11,21].

#### 3.2.4. The NAVOTEL System

The NAVOTEL system (Nose, Adenoids, Velum, Oropharynx, Tongue, Epiglottis, Larynx) was developed to address the limitations of the VOTE classification by expanding the number of evaluated anatomical sites [25]. In addition to the standard VOTE scale, the nasal cavity (Nose), adenoids (Adenoids), and the larynx/supraglottis (Larynx) were included. Studies have demonstrated a significant correlation between NAVOTEL scores and OSA severity. NAVOTEL is considered a comprehensive system suitable for documenting surgical indications in pediatric DISE.

#### 3.2.5. The PedDISE-8 System

The PedDISE-8 system is a more recent classification tool that evaluates eight surgically relevant sites of airway obstruction independently in pediatric patients. It is distinguished by a detailed differentiation of closely situated pathologies that may require different treatment (e.g., it distinguishes the tongue base from the lingual tonsil). Validation studies have demonstrated good internal and external validity, with the number of sites exhibiting severe obstruction correlating positively with obstructive AHI [19].

#### 3.2.6. The International Pediatric Sleep Endoscopy Scale

The International Pediatric Sleep Endoscopy Scale [47] is a recently developed classification system with the aim of standardizing DISE assessment in children. The scale evaluates the maximum degree of airway obstruction at seven key anatomical sites identified by expert consensus as clinically relevant for surgical intervention: the nasal airway, nasopharynx/adenoid pad, velum (soft palate), lateral oropharyngeal walls/palatine tonsils, tongue base, epiglottis, and arytenoids. IPSES was designed as a universal pediatric DISE assessment tool and represents a step toward improving the consistency and quality of diagnostic evaluation and treatment planning for pediatric OSA. Its primary objective is to standardize documentation, thereby facilitating prospective multicenter studies and robust meta-analyses evaluating the outcomes of DISE-directed surgical interventions.

### 3.3. Anesthetic Protocols in Pediatric DISE

Among available agents, propofol and dexmedetomidine (DEX) are most commonly recommended for sedation during pediatric DISE. Propofol has historically been the most frequently used anesthetic agent due to its rapid onset, short duration of action, and ease of titration. Despite these advantages, propofol does not replicate natural sleep architecture and is associated with dose-dependent suppression of upper airway muscle tone, which may lead to an overestimation of airway obstruction [6]. DEX has gained increasing acceptance as a preferred agent for pediatric DISE because it induces a sedative state that closely resembles NREM sleep [6,48]. Compared with propofol, DEX exerts a more favorable effect on upper airway patency and is associated with a lower incidence of hypoxemia and desaturation [6,49,52]. However, its use is limited by a relatively slow onset of action and the potential for bradycardia and hypotension. The combination of DEX and ketamine (DK) is frequently considered an optimal anesthetic regimen for pediatric DISE [6]. Ketamine is a dissociative anesthetic that preserves spontaneous respiration and airway reflexes and may counteract the hemodynamic side effects of DEX, such as bradycardia or hypotension. Nonetheless, concerns have been raised that ketamine may increase upper airway muscle tone, potentially masking the true pattern of airway collapse and thereby affecting diagnostic accuracy [6,11]. Inhalational anesthetic agents, such as sevoflurane, are commonly used in pediatric anesthesia for induction and establishment of intravenous access. However, these agents should be discontinued prior to the diagnostic phase of DISE, as they may exaggerate airway collapse in a dose-dependent manner and distort endoscopic findings [6,11].

To minimize the influence of anesthetic agents on upper airway dynamics, structured procedural protocols have been developed. One such approach is the “on–off” technique using sevoflurane, implemented as part of a quality improvement initiative in pediatric centers [48]. This method involves brief administration of sevoflurane and nitrous oxide at the beginning of the procedure to facilitate intravenous cannulation and atraumatic endoscope insertion, followed by immediate discontinuation of inhalational agents. The diagnostic phase of DISE is then performed after administration of a DEX bolus, thereby reducing anesthetic interference with airway behavior [48]. Accurate assessment of sedation depth is another key determinant of diagnostic reliability. Expert consensus recommends clinical evaluation based on the presence of snoring or obstructive breathing patterns in conjunction with a lack of response to endoscope manipulation [6]. Bispectral index (BIS) monitoring is sometimes used as an adjunct, with target values typically ranging from 60 to 75; however, its validity in pediatric patients has not been fully established [6,11]. Certain anesthetic agents commonly used in pediatric practice are strongly discouraged during DISE because of their potential to compromise diagnostic accuracy and patient safety. Opioids, such as remifentanil, may induce respiratory depression and excessive upper airway collapse, preventing simulation of natural sleep [6]. Similarly, benzodiazepines, particularly midazolam, should be avoided as premedication, as they may exacerbate airway obstruction, suppress REM sleep, and significantly alter endoscopic findings [6]. In addition, the use of topical anesthetics and nasal decongestants is not recommended during diagnostic DISE, as these agents may modify upper airway dynamics and increase the risk of laryngospasm [6,11]. A summary of the anesthetic agents used is presented in Table 3. Overall, anesthetic management in pediatric DISE requires close collaboration between the anesthesiologist and the surgeon.

### 3.4. Surgical Outcomes in Selected Pediatric Patient Groups

The role of DISE in pediatric OSA has expanded from a purely diagnostic modality to a critical component of individualized surgical planning, particularly in complex or treatment-refractory cases. Although AT remains the first-line therapy, persistent OSA is observed in approximately 10–20% of otherwise healthy children and in a substantially higher proportion of patients with comorbidities such as obesity or Down syndrome [29]. Multiple studies have demonstrated that DISE frequently alters surgical decision-making. In a cohort study by Frederick et al. [29], involving 106 children—both surgically naïve and previously treated—DISE performed during primary surgery identified multilevel obstruction in 10.4% of patients. The most common sites of collapse were the tongue base (32.1%), soft palate (21.7%), and epiglottis (13.2%). Consequently, DISE findings prompted additional procedures beyond AT, including pharyngoplasty (27.4%), tongue base reduction (20.1%), and supraglottoplasty (5.7%). A systematic review by Saniasiaya and Kulasegarah [33], involving 996 pediatric patients, reported that DISE modified surgical management in approximately 30% of cases. In children with persistent OSA following AT, DISE consistently identified at least one site of obstruction, most commonly at the tongue base, lingual tonsils, or epiglottis [36].

#### 3.4.1. Surgically Naïve Children with Small Palatine Tonsils

Children with OSA and small palatine tonsils (Brodsky grade 1+) represent a distinct subgroup in whom standard AT may be ineffective. In a cohort of 40 such patients, Williamson et al. demonstrated that the most frequent sites of obstruction identified by DISE were the adenoids (72.5%) and tongue base or lingual tonsils (52.5%), whereas palatine tonsils contributed to obstruction in only 25% of cases. DISE-guided management helped avoid unnecessary tonsillectomy in most patients, favoring adenoidectomy or supraglottoplasty instead. This approach resulted in a significant reduction in AHI from 14.4 to 8.0 events per hour [36].

#### 3.4.2. Children with Down Syndrome (Trisomy 21)

Children with Down syndrome are at high risk of AT failure due to anatomical and functional factors, including macroglossia, generalized hypotonia, and midface hypoplasia. Akkina et al. [44] evaluated DISE-directed surgical outcomes in children with Down syndrome, both before and after AT. In patients with prior AT, the tongue base and supraglottis were the most common sites of obstruction. Although targeted surgical interventions resulted in improvements in polysomnographic parameters, these changes often failed to reach statistical significance, underscoring the challenges of achieving complete resolution of OSA in this population despite DISE-guided management.

#### 3.4.3. Children with Laryngomalacia

DISE is particularly valuable in the diagnosis of sleep-dependent or occult laryngomalacia, which may not be evident during awake examination. In a study of children under two years of age, Love et al. [38] found that 56.1% of patients with DISE-confirmed laryngomalacia did not require AT and instead underwent supraglottoplasty. This intervention led to a 78% reduction in AHI, highlighting the role of DISE in avoiding unnecessary tonsillar surgery in very young children.

### 3.5. Outcomes of Selected DISE-Directed Surgical Procedures

#### 3.5.1. Lingual Tonsillectomy

Lingual tonsil hypertrophy is a frequent cause of persistent pediatric OSA. In a large retrospective study of 174 patients, Williamson et al. evaluated the safety and efficacy of lingual tonsillectomy performed as part of DISE-directed multilevel surgery. Postoperatively, mean AHI decreased from 18 to 3 events per hour, with complete surgical success (AHI < 5) achieved in 82.8% of patients. The procedure demonstrated an acceptable safety profile, with a postoperative bleeding rate of 6.9% and a readmission rate of 2.3%, even in a population with significant comorbidities [32].

#### 3.5.2. Simultaneous Lingual Tonsillectomy and Epiglottopexy

Epiglottic collapse often coexists with tongue base obstruction. Maksimoski et al. assessed outcomes of simultaneous lingual tonsillectomy and epiglottopexy in 24 children, half of whom had Down syndrome. The median reduction in obstructive AHI was 69.9%, and overall surgical success was achieved in 84.6% of patients. Importantly, no new cases of postoperative dysphagia were reported, suggesting that this combined approach is both effective and safe in selected complex cases [43].

## 4. Discussion

While AT remains the first-line treatment for children with OSA associated with adenotonsillar hypertrophy, the prevalence of residual or persistent OSA following surgery remains substantial, ranging from approximately 30–75% in high-risk populations [5,11,39]. In this context, DISE plays an important role in identifying multilevel airway obstruction that may underlie primary treatment failure. Multiple studies have demonstrated that DISE alters the planned surgical approach in approximately 30–60% of pediatric patients, indicating that reliance on awake examination alone may lead to suboptimal therapeutic decision-making [29,32,33].

One of the principal barriers to the advancement of DISE research and the comparability of outcomes across institutions is the absence of a universally accepted classification system [2,47]. Adult-derived systems like VOTE omit critical pediatric structures such as the adenoids and nasal cavity, prompting the development of pediatric-specific tools [11,25]. In response to these limitations, several pediatric-specific classification systems have been developed, including the Chan–Parikh scale, SERS, and the NAVOTEL system [21,25]. The recent introduction of the IPSES addresses these anatomical gaps, notably distinguishing the tongue base from the epiglottis to identify specific collapse mechanisms like the ‘trapdoor’ phenomenon [26,47]. Validation studies have demonstrated moderate interobserver reliability, with kappa values between 0.43 and 0.57, and the highest agreement observed for the soft palate. In day-to-day clinical practice, this moderate level of agreement suggests that subjective interpretation remains a prominent factor, even when utilizing a standardized framework. This variability has direct implications for surgical planning; differing interpretations of airway collapse severity could lead to disparate surgical recommendations for the same patient. Therefore, clinicians must interpret scores cautiously, integrating them with the patient’s broader clinical picture and relying on institutional consensus to guide surgical decisions. Adoption of a unified classification system, such as IPSES, is therefore essential to enable multicenter studies, improve reproducibility, and establish evidence-based treatment algorithms in pediatric OSA [47].

Appropriate anesthetic management is an important component of pediatric DISE, as it directly influences the reliability and interpretability of endoscopic findings [6]. The primary goal of sedation during DISE is to reproduce physiological sleep as closely as possible while ensuring patient safety, procedural tolerance, and stable cardiorespiratory function. Ideally, sedation should allow rapid and predictable achievement of the target depth of sleep without artificially exaggerating upper airway collapse beyond that observed during natural sleep. However, it should be acknowledged that no currently available anesthetic agent is capable of reproducing REM sleep, a phase during which airway obstruction may be more pronounced in children [6]. The literature describes considerable heterogeneity in anesthetic techniques used for pediatric DISE. Nevertheless, expert consensus consistently emphasizes the importance of minimizing pharmacologically induced muscle relaxation and respiratory depression. The selection of an appropriate anesthetic protocol for pediatric DISE remains a subject of ongoing debate. No single drug truly reproduces natural, physiological sleep. Most notably, no currently available agent is capable of replicating rapid eye movement (REM) sleep, a phase during which airway obstruction is frequently most pronounced. While dexmedetomidine is increasingly favored because the sedative state it induces closely approximates NREM sleep, it is not a perfect solution; its use is limited by a delayed onset of action and the potential for hemodynamic side effects, such as bradycardia and hypotension. Because every anesthetic technique inherently compromises some aspect of natural airway dynamics, the question of the ‘ideal’ protocol is far from settled, requiring clinicians to carefully weigh the benefits and limitations of their chosen regimen. Current evidence indicates that propofol and DEX are the two most commonly utilized agents in this setting [6,49]. Propofol is widely favored due to its rapid onset, ease of titration, and short recovery time. However, it does not replicate natural sleep architecture and is associated with dose-dependent reductions in upper airway cross-sectional area, potentially leading to an overestimation of obstruction severity [6]. In addition, propofol use has been linked to a higher incidence of hypoxemia and oxygen desaturation compared with DEX. Adult studies report lower minimum oxygen saturation and higher hypoxemia risk with propofol, and suggest that DEX offers improved respiratory stability and higher operator satisfaction, albeit with a higher rate of sedation failure [52]. These effects may complicate interpretation of DISE findings, particularly in infants and young children, in whom an increased susceptibility to airway obstruction has been observed [6]. DEX more closely mimics NREM sleep and exerts a lesser suppressive effect on upper airway muscle tone, making it an attractive agent for pediatric DISE [6]. Its primary limitations include a delayed onset of action and the risk of bradycardia and hypotension. Notably, one study reported bradycardia (heart rate < 60 beats/min) in 58.3% of patients receiving DEX; however, these events were transient and managed without significant clinical consequences [6,11]. DK has been proposed as an optimal anesthetic regimen for pediatric DISE [6]. Ketamine preserves spontaneous respiration and airway reflexes and may counterbalance the hemodynamic effects of DEX. Comparative studies have demonstrated a significantly lower incidence of oxygen desaturation below 85% in patients receiving this combination compared with propofol alone (3.1% vs. 38.5%) [6]. Nevertheless, concerns persist that ketamine-induced increases in muscle tone may obscure the true pattern of airway collapse, potentially reducing diagnostic accuracy [11].

DISE refines treatment plans in 30–60% of pediatric patients, avoiding ineffective procedures and guiding targeted interventions such as supraglottoplasty in infants [33]. For instance, in surgically naïve children with small palatine tonsils (Brodsky grade 1+), DISE prevents unwarranted tonsillectomies by identifying alternative obstruction sites like the tongue base or epiglottis [36]. While evidence linking DISE to consistently superior polysomnographic outcomes remains limited in adults [12], tailored surgery in children with complex or refractory OSA yields meaningful improvements in AHI and oxygen saturation [11,32], offering a safe and effective alternative to long-term CPAP therapy. Despite these benefits, achieving complete disease normalization (AHI < 1 event/h) remains challenging, particularly in patients with greater baseline severity [38]. Several subgroups face a high risk of residual OSA despite optimized, DISE-guided management. In obese children, airway collapse patterns vary [20], but a higher prevalence of concentric pharyngeal collapse [50] and severe obesity (BMI z-scores > 2) predict poorer surgical outcomes [32]. Similarly, children with Down syndrome frequently present with multilevel obstruction requiring targeted procedures like lingual tonsillectomy or epiglottopexy; however, intrinsic hypotonia and craniofacial characteristics act as independent risk factors for persistent disease [32,44].

It is critical to acknowledge that pediatric patients undergoing DISE are rarely straightforward clinical cases. The cohorts evaluated in the literature frequently include children with persistent OSA, obesity, Down syndrome, craniofacial differences, or a history of previous upper airway surgeries. These baseline characteristics act as significant confounding variables. Consequently, while polysomnographic improvements are consistently observed following DISE-directed multilevel surgery, these positive outcomes cannot be solely attributed to the application of DISE itself. The intrinsic severity of the underlying syndromic or anatomical abnormalities independently influences both the surgical success rate and the overall trajectory of the disease.

Emerging applications of DISE include its use in CPAP titration (DISE-PAP) and in the evaluation of CPAP intolerance, thereby allowing identification of pressure-induced collapse mechanisms, such as epiglottic “trapdoor” obstruction [16]. Additionally, integration of advanced imaging modalities, including optical coherence tomography and cine magnetic resonance imaging, may further enhance characterization of airway dynamics and complement endoscopic findings in future diagnostic paradigms [27,31].

## 5. Conclusions

Drug-induced sleep endoscopy has emerged as a highly promising and valuable adjunct in the evaluation of complex and persistent pediatric OSA, helping to bridge the diagnostic gap between awake clinical examination and PSG. By enabling dynamic visualization of the upper airway under conditions that approximate natural sleep, DISE allows for a precise identification of obstruction sites and mechanisms, thereby facilitating individualized surgical planning. In children with persistent OSA following AT, small palatine tonsils, craniofacial anomalies, Down syndrome, or suspected sleep-dependent laryngomalacia, DISE provides clinically actionable information that frequently alters management and helps avoid unnecessary or ineffective procedures. Pediatric-specific classification systems—particularly the International Pediatric Sleep Endoscopy Scale—represent a critical step toward standardized documentation and improved comparability of outcomes across centers. Despite its demonstrated diagnostic value, the interpretation of DISE remains inherently subjective and influenced by anesthetic technique. Consequently, the widespread adoption of standardized assessment tools and optimized sedation protocols is strongly encouraged. While the current evidence demonstrates that DISE provides clinically actionable information that frequently alters surgical management, it is important to recognize the limitations of the existing, primarily retrospective data. The current body of literature does not yet support definitive claims regarding the consistent, long-term superiority of these tailored interventions. Therefore, future research must focus on well-designed, prospective, multicenter studies to determine whether DISE-directed surgical strategies actually yield superior long-term polysomnographic and patient-reported clinical outcomes compared with conventional management [11].

## 6. Limitations

This review has several limitations. This is a structured narrative review. A formal risk-of-bias assessment was outside the current scope. Our literature search was restricted solely to the PubMed database. Most pediatric studies evaluating DISE are retrospective and single-center in design. There is a risk of overlapping patient populations. Therefore, readers should interpret the efficacy of these surgical interventions with caution. Interpretation of DISE findings remains partly subjective and operator-dependent, even when standardized scoring systems are used. Additionally, pharmacologically induced sleep does not fully replicate physiological sleep, particularly due to the absence of REM sleep, which may influence observed airway behavior.

## Figures and Tables

**Table 1:** Comparison of all studies.

Author	Study Design	Patients (N)	Patient Age	Previous Surgery Info	Anesthetic Protocol	Scoring System	Follow-Up	Type of Article	Main Findings
Wang et al. [4]	Systematic review and meta-analysis	761	Ranged from approximately 4.1 to 6.2 years across the included studies	All pediatric patients had no history of upper airway surgery, craniofacial abnormalities, or syndromes other than obstructive sleep apnea–hypopnea syndrome	Across the included studies, pediatric patients undergoing DISE were typically sedated using either a continuous intravenous infusion of propofol or dexmedetomidine as the most common sedation options. Specific agents utilized in the pooled studies included propofol, isoflurane, remifentanil, and dexmedetomidine	Not specified	Ranged from 3 weeks to 1 year depending on the specific included study	Systematic Review and Meta-Analysis	In pediatric patients with OSAHS, obstruction rates observed during DISE were 93% for the nasopharynx (adenoid), 35% for the soft palate, 76% for the oropharynx (tonsils), 32% for the tongue base, 31% for the supraglottic level, and 60% for multi-level obstruction. Postoperative symptoms and sleep-related parameters improved significantly compared to preoperative values, with DISE findings potentially enhancing surgical success rates and avoiding unnecessary procedures
Cousineau et al. [5]	Nation-wide online cross-sectional survey	Not applicable	Not applicable	Not applicable	There was no agreement regarding the optimal anesthetic agent(s) to use during DISE, though propofol and dexmedetomidine were the most frequently used agents by respondents (58.3% and 50.0%, respectively)	None of the scoring systems listed in the survey were consistently used by participants, and 41.7% (5/12) of them did not use any scoring system	Not specified	Original Research Article	The survey highlighted a lack of overall consensus (55% agreement) among Canadian otolaryngologists regarding the management of pediatric obstructive sleep apnea and DISE. While there was consensus on clinical evaluation methods and airway sites examined during DISE, there was no consensus regarding the ideal anesthetic protocol, scoring systems, or the routine use of polysomnography prior to surgery.
Liu et al. [6]	Retrospective literature review	1110	2 months to 19 years	Five studies evaluated pre-TA, two evaluated post-TA, and four evaluated both pre- and post-TA (one study evaluated Prader–Willi syndrome patients regardless of OSA diagnosis)	Eleven out of twelve studies used a sevoflurane inhalational induction for IV line placement and mostly transitioned to a total intravenous anesthetic (TIVA) for maintenance. Maintenance regimens varied: seven studies used propofol alone (bolus, intermittent boluses, or infusion), three studies used a combination of remifentanil and propofol infusions, one study used a dexmedetomidine bolus alone, one used sevoflurane alone, and one compared different regimens (propofol, sevoflurane–propofol, and dexmedetomidine–ketamine)	Not applicable	Not specified	Review article	A wide variety of anesthetic techniques are used for pediatric DISE, but there is a lack of consensus guidelines or high-quality protocols. The combination of ketamine and dexmedetomidine may offer the most favorable profile with minimal negative effects on airway dynamics, whereas premedication, inhalational agents, and opioids tend to worsen upper airway obstruction
Raposo et al. [7]	Retrospective case series	56	Surgically naïve group: 6.48±4.69 years; After adenotonsillectomy group: 8.67±4.90 years	23 children were surgically naïve (with risk factors for persistent obstructive sleep apnea), and 33 children had persistent obstructive sleep apnea after adenotonsillectomy	Sedation was induced by inhaled sevoflurane until intravenous access was obtained; then, sevoflurane was stopped and intravenous propofol administered, followed by an infusion	Chan–Parikh system	Not specified	Original research	DISE findings differed between surgically naïve children (most common obstruction sites were adenoids at 78.2% and lateral pharyngeal walls/tonsils at 82.6%) and children with persistent obstructive sleep apnea after adenotonsillectomy (most common sites were adenoids at 54.5%, supraglottis at 48.5%, and tongue base at 45.5%). No correlation was found between obstructive apnea–hypopnea index and DISE findings, but increased obstruction at the tongue base and the presence of multilevel obstruction predicted a lower saturation nadir in children with persistent obstructive sleep apnea after adenotonsillectomy.
Kirkham et al. [8]	Retrospective comparative cohort study	117	Mean 6.5 ± 4.0 years (range 1–18 years)	41% had previous tonsillectomy. Excluded history of other upper airway or tracheal surgery (except previous AT).	Inhaled sevoflurane induction. Maintained intravenously with either propofol infusion (*n* = 67) or dexmedetomidine infusion (*n* = 50) with/without ketamine	7 separate scoring systems	Retrospective billing and PSG database match	Comparative Clinical Research	Indicates that anesthetic choice (propofol vs. dexmedetomidine) does not introduce clinically significant variability in DISE findings.
Ma et al. [9]	Retrospective cohort study	86	Children aged 3 years and younger	Patients were excluded if they underwent any upper airway surgery prior to their DISE procedure.	DISE was performed in the operating room by a single pediatric otolaryngologist with intravenous propofol titration by a pediatric anesthesiologist	Recorded findings were evaluated using a validated 10-site scoring system.	Information regarding the performance of additional procedures following the DISE procedure was collected until January 2023.	Original Research	Multisite airway obstruction is common among children with OSA, with the most common sites of significant obstruction being the aryepiglottic folds. There was no statistically significant difference in the total number of sites of obstruction or the frequency of obstruction sites between infants and toddlers
Blokland et al. [10]	Retrospective cohort study	19	Pediatric cohort (unspecified range; high clinical complexity)	15 of 19 (78.9%) had prior upper airway surgery: 12 prior AT, 2 prior adenoidectomy, 1 prior tonsillectomy; 4 were surgically naïve.	No fixed protocol. Typically avoided premedication; used IV dexmedetomidine (0.5–1 µg/kg load, 0.5–1 µg/kg/h infusion) and propofol (TCI Paedfusor, 2 µg/kg/min). Sevoflurane induction if needed	VOTE score used; no single standardized grading system across surgeons.	Pre- and post-operative PSG comparison	Original Clinical Research	DISE-directed surgery successfully guided targeted, multilevel interventions. Significant improvements in OSA severity were seen in younger, non-obese children without Trisomy 21 (OAHI improved from 10.1 to 2.2 in severe OSA, and 8.2 to 2.6 in non-obese children).
Baldassari et al. [11]	Expert Consensus Statement	Not applicable	0–18 years	The consensus statements address both “surgically naïve” children and children with persistent OSA following prior adenotonsillectomy	The development group agreed that either propofol or dexmedetomidine is an optimal form of sedation for pediatric DISE. Inhalational agents may be used for induction and to gain intravenous access during DISE but should be discontinued for the diagnostic portion of the study. The surgeon and anesthetist should agree on the sedation protocol prior to DISE, and the level of sedation should be titrated to audible snoring, an obstructive breathing pattern, or both, while allowing for flexible endoscopy without patient reactivity or awakening	The expert panel agreed that during pediatric DISE, the following parameters should be observed and documented for each anatomic level: (a) site, (b) pattern or shape, and (c) severity of obstruction. The anatomic sites that should be assessed and documented include the nasal (including nasal cavities, nasopharynx), palate/velum, pharyngeal airway (including lateral oropharyngeal wall and tongue base), and supraglottic larynx	Not specified	Expert Consensus Statement	Expert consensus was achieved for 26 statements pertaining to indications, protocols, and outcomes for pediatric DISE. Polysomnography is recommended prior to DISE to confirm the presence and severity of OSA, and DISE has limited utility in children with an apnea–hypopnea index (AHI) < 2.
Sims et al. [14]	Systematic review and meta-analysis	283	Pediatric patients	All patients had prior adenotonsillectomy with persistent or recurrent obstructive sleep apnea.	Not specified in the text	Not specified in the text	Not specified in the text	Systematic Review and Meta-Analysis	DISE-directed tongue surgery in children with persistent obstructive sleep apnea can reduce the AHI by approximately 50%. Lowest oxygen saturation improved by nearly 3%
Sigaard et al. [15]	Systematic review	376	Under 18 years old	Included studies consisted of both surgically naïve children and those with persistent OSA following prior upper airway surgery	The text notes that most studies used inhalation followed by propofol maintenance for sedation during the procedure	The included studies utilized varying scoring systems, most commonly the Chan scoring system or custom frameworks assessing fixed and dynamic obstructions across various airway levels	The follow-up periods varied, but were noted by the authors to be short, with some studies having a follow-up of less than 6 months	Systematic review	Based on DISE findings, surgeons changed the planned surgical strategy in an average of 50.4% of the patients.
Arganbright et al. [18]	Review Article	Not applicable	Not explicitly specified	Discusses patients with persistent obstructive sleep apnea (OSA) following adenotonsillectomy (AT), as well as surgically naïve patients.	The text notes that most children require an inhalational agent before intravenous (IV) line insertion, while topical nasal anesthetics and decongestants are avoided to prevent altering airway reflexes and inferior turbinate evaluations. Commonly proposed agents include propofol, midazolam, dexmedetomidine (DEX), ketamine, and sevoflurane, though no single protocol has been universally accepted	The review outlines multiple published scoring systems used in pediatric DISE without endorsing a single one, including VOTE, Chan, Sleep Endoscopy Rating Scale (SERS), Bachar, Boudewyns, Fishman, and a system by Williamson et al.	Not specified	Review article	Pediatric DISE has expanded from evaluating persistent OSA after adenotonsillectomy (AT) to assessing certain surgically naïve patients (such as those at high risk or with small tonsils/adenoids), DISE is effective at identifying obstruction sites that awake exams miss, but a major limitation in the field remains the lack of a universally accepted anesthetic protocol and standardized scoring system.
Jaffal et al. [19]	Retrospective chart review	86	Median 3.5 years (IQR 2.4–5.3, range 0.02–17.1 years)	18.6% prior adenoidectomy, 12.8% prior tonsillectomy, 1.2% prior inferior turbinate reduction, 1.2% prior supraglottoplasty.	Inhaled sevoflurane or IV propofol induction, maintained with IV propofol. Exam initiated after sevoflurane was discontinued and the patient was breathing spontaneously	PedDISE-8	Retrospective chart database review	Instrument Validation and Clinical Research	PedDISE-8 grading system demonstrated moderate to good inter-rater (ICC 0.67–0.88) and intra-rater (ICC 0.71–0.87) reliability across all eight sites. External validity was confirmed as the number of severely obstructed sites positively correlated with oAHI (τ = 0.119, *p* = 0.004).
Lookabough et al. [20]	Prospective, observational cohort study	51	2–12 years old	Surgically naïve children	After IV placement sevoflurane and nitrous oxide were discontinued. A loading dose of dexmedetomidine was administered as a slow bolus. Ketamine was then administered immediately after starting the dexmedetomidine bolus	Chan–Parikh system	Pre- and postoperative polysomnograms were gathered as available	Original Article/Prospective Observational Cohort Study	There was no statistically significant difference in airway obstruction at the velum, adenoid, lateral pharyngeal walls, tongue base, or supraglottis between obese and non-obese children. Obese children were not significantly more likely to have multi-level obstruction compared to non-obese children.
Williamson et al. [21]	Technical Report	Not specified in the text	Not specified in the text	Not specified in the text	Anesthesia is first induced using an inhaled anesthetic agent, allowing for intravenous access to be obtained. The inhalation agent is then discontinued and a propofol infusion is used	The proposed DISE scoring system records the operator’s evaluation of the bilateral nasal airway, adenoid, retropalatal airway, oropharyngeal airway, retrolingual airway, and laryngeal airway separately. Obstruction at each site or by the specified structure is broadly categorized as non-obstructive, partially obstructive, or significantly obstructive.	Not specified in the text	Open Access Technical Report	The scope of DISE research is historically limited by a lack of consistent quantitative data, uniform techniques, and standardized scoring systems among institutions. The report proposes a standardized protocol and scoring system for pediatric DISE that comprehensively addresses all levels of potential upper airway obstruction.
Williamson et al. [22]	Intra-rater test–retest and inter-rater reliability analysis	30	14 years old and younger	The reviewers were blinded to the medical and surgical history of the patients undergoing DISE	DISE was performed in the operating room with propofol sedation titrated and monitored by the anesthesia team following a standard protocol described in a previously published technical report	The videos were graded using both the VOTE classification system and a novel, internally developed pediatric DISE scoring system.	The study focuses on evaluating the reliability of scoring systems based on recorded videos rather than longitudinal patient follow-up; the second review of the videos by the raters was completed at least one week after their first review.	Original Article	The novel pediatric scoring system demonstrated moderate to near-perfect intra-rater (test–retest) agreement, with weighted kappa coefficients of 0.62 and 0.87 at the 25th and 75th percentiles, respectively. The novel scoring system demonstrated comparable reliability to the established VOTE system while evaluating a more comprehensive set of potential airway obstruction sites pertinent to pediatric obstructive sleep apnea.
Iannella et al. [24]	A web-based cross-sectional international survey	Not applicable	Not applicable	Not applicable	According to the experts’ responses, propofol was the most frequent drug used when performing DISE in children (67.1%), followed by Dexmedetomidine (25.6%), Ketamine (4.9%), and Midazolam (2.4%). Furthermore, 67.4% of the interviewed experts reported using the Bispectral Index (BIS) during pediatric DISE	The VOTE classification was the most frequently used scoring system during DISE (65.1%), followed by NOHL (15.7%), descriptive reports (15.7%), and the Chan–Parikh classification system (3.6%)	Not applicable	Article	Only 63.4% of the interviewed experts reported that they perform DISE in pediatric OSA patients. A large majority (81.9%) of responders agreed that DISE should be the first diagnostic step in children with persistent OSA after adenotonsillectomy surgery.
Qarbal et al. [25]	Single-center prospective cohort study	99	Mean 7.23 ± 3.73 years (median 6.7, range 0.2–17.9 years)	High complexity; details of prior surgery not fully specified, but all had pathological preoperative sleep recordings.	Standardized: naso-buccal mask induction with sevoflurane followed by intravenous maintenance with propofol; no local anesthesia used	NAVOTEL and VOTE	Preoperative PSG (27%) or polygraphy (73%) compared with clinical outcomes.	Original Clinical Research	NAVOTEL was exhaustive for pediatric surgical planning, capturing sites omitted by the adult VOTE system (e.g., adenoids, nasal cavity, sleep laryngomalacia). NAVOTEL scores correlated with OAHI severity (ρ = 0.2; *p* = 0.04) and added 50 surgical procedures compared to awake exam.
Parikh et al. [26]	Expert consensus statement	Not applicable	Not applicable	Not applicable	Not applicable	Not applicable	Not applicable	Expert Consensus Statement	The International Pediatric Otolaryngology Group (IPOG) formally quantified and developed consensus statements to help standardize the scoring and documentation of DISE. Experts agreed that severity should be based on the maximal obstruction observed at specific anatomical sites and graded on a 3-point scale (<50%, 50–89%, ≥90%).
Goshtasbi et al. [27]	Proof-of-concept study	2	7 and 12 years old	Patient one had persistent sleep-disordered breathing after previously undergoing an adenotonsillectomy. Patient two could not tolerate a sleep study or physical exam, and the text does not indicate any previous surgery for this individual.	Article does not describe the specific pharmacological anesthetic protocol	VOTE system	After the procedure, the first patient underwent conservative management and had a 6-month sleep study, whereas the second patient underwent adenotonsillectomy	Preliminary Report	The proof-of-concept study successfully demonstrated that long-range optical coherence tomography (LR-OCT) can be used to evaluate the pediatric airway during DISE. The LR-OCT cross-sectional images allowed for the successful creation of three-dimensional (3D) volumetric reconstructions of the patients’ airways.
Adler et al. [28]	Retrospective cohort/records review	12	Pediatric cohort (range 2–18 years, including syndromic cases)	Many had prior tonsillectomy and/or adenoidectomy, or complex airway interventions.	Inhalational sevoflurane induction titrated down until return of upper airway reflexes, then CPAP titration under flexible fiberoptic view at the velum. No premedication.	VOTE and SERS scores calculated.	Pre- and post-operative PSG (within 6 months)	Original Clinical Research/Case Series	DISE-observed CPAP titration successfully identified areas of dynamic collapse (most severe at velum and oropharynx) and optimized positive pressure settings in 7/12 patients. Provided an alternative for children non-compliant with standard sleep study titrations.
Frederick [29]	Retrospective chart review	106	Ranging from 1 to 18 years, with a mean age of 8.4 years (SD 4.57)	All included patients were surgically naïve prior to the studied intervention	The text does not detail the specific pharmacological agents or the exact anesthetic protocol used during the procedure	The document does not explicitly name a formal, standardized scoring system	The mean length of time between the surgery and the postoperative polysomnogram (PSG) was 4.2 months (SD 2.8)	Original Research	DISE is highly effective in intraoperative decision-making for surgically naïve pediatric patients with severe sleep apnea. Surgical success—defined as an oAHI decrease by at least one diagnostic category—occurred in 76.4% of patients.
Trandafir et al. [30]	Retrospective case series	123	Median age at the time of surgery was 8 years	50 patients (41%) had a history of prior upper airway surgery, consisting of tonsillectomy and/or adenoidectomy	After the induction of anesthesia using inhalational agents (Sevoflurane), an intravenous access was established. Sevoflurane was then discontinued, and the procedure only commenced once the expirated Sevoflurane reached zero. Sedation was achieved using an intravenous propofol infusion (10–15 mg/kg/h) along with a bolus of 1 to 2 mg/kg. The level of sedation during the Drug-Induced Sleep Endoscopy (DISE) was titrated based on clinical parameters—specifically audible snoring, the presence of an obstructive pattern, and unresponsiveness to the introduction of the flexible scope—in conjunction with targeting a bispectral index between 50 and 60.	Study does not name a specific standardized scoring or grading system used to quantify the obstruction	The postoperative respiratory polygraphy (PG) was performed between 3 and 12 months after the surgery, with a mean delay of 7 months. Complications were tracked until Postoperative Day (POD) 30	Original Research	Lingual tonsillectomy (LT), when performed as part of a DISE-directed multilevel upper airway surgery, is highly efficient and safe for treating complex pediatric OSA. The procedure resulted in a dramatic decrease in the median Apnea–Hypopnea Index (AHI), dropping from 18 events/h preoperatively to 3 events/h postoperatively (*p* < 0.001)
Li et al. [31]	Retrospective cohort study	137	Mean age was 10.4 years	Patients who had not previously undergone adenoidectomy and/or tonsillectomy were excluded. Additionally, those with a history of open airway reconstruction surgery were also excluded.	A pharmacologic sleep state was then induced using a loading dose of dexmedetomidine (2 mcg/kg) over 10 min, along with a bolus of ketamine (1 mg/kg). Following this loading dose, dexmedetomidine was administered via continuous infusion (2 mcg/kg/h) until the end of the procedure.	Internal rating scale	The study focuses on the diagnostic findings from same-day sequential DISE and cine MRI evaluations. The article does not describe longitudinal postoperative follow-up intervals.	Original Article (Retrospective cohort study)	DISE is more sensitive to partial versus complete collapse and provides a superior evaluation of nasal and supraglottic obstruction. Cine MRI offers better soft tissue resolution for lymphoid tissue hypertrophy and provides a global view of primary and secondary airway obstruction. The two modalities are complementary in evaluating persistent pediatric OSA.
Williamson [32]	Retrospective case series study	174	The mean age of the patients was 8.29 ± 3.49 years, with a range of 1.89 to 15.62 years	Within the study group, 116 patients (66.7%) had a previous history of adenoidectomy, 99 patients (56.9%) had a previous history of tonsillectomy	DISE was performed in the operating room using propofol sedation, which was titrated and monitored by the anesthesia team following a standard protocol. Following this, general endotracheal anesthesia was employed by a pediatric anesthesiologist for the surgical procedure. Marcaine 0.25% with epinephrine 1:200,000 was injected into the right and left tongue base to provide postoperative local anesthesia and reduce the need for intravenous narcotics in the recovery room.	DISE findings were interpreted using a previously published scoring system noted for good inter- and intrarater reliability, though the specific name of the scoring system is not explicitly stated in the text.	Patients with moderate to severe obstructive sleep apnea (OSA) or persistent symptoms were advised to complete a postoperative polysomnography (PSG) no sooner than 90 days after surgical intervention	Original Research	DISE-directed lingual tonsillectomy in multilevel sleep surgery demonstrated a promising surgical success rate of 82.8%. Surgical failure, defined as a postoperative apnea–hypopnea index (AHI) of 5 or greater, occurred in 17.2% of patients.
Saniasiaya et al. [33]	Systematic review	996	Between 4 and 9 years, with a mean age of 6 years	The majority of the patients (872 patients, representing 87.6%) were surgically naïve	A combination of sevoflurane and propofol was used in three studies, ramifentanyl and propofol in one study, and a ketamine and dexmedetomidine combination in two studies, while one study did not mention the specific anesthetic agent used. The ideal moment to start the airway assessment during the DISE procedure is determined with the help of the Bi-spectral index (BIS), which objectively measures the depth of sedation	Chan–Parikh system was utilized in three studies, a generic DISE scoring system in one study, and the scoring system utilized was not mentioned in the remaining three studies	Repeated polysomnography (PSG) post DISE-directed surgery was carried out in three studies. Additionally, one study evaluated the Respiratory Disturbance Index (RDI) and oxygen saturation nadir at one-year post-operatively	Review Article (Systematic Review)	DISE-directed surgery was found to be an effective and safe therapeutic approach for treating pediatric obstructive sleep apnea. Surgical management plans were changed in 295 patients (30%) following DISE. The majority of patients (86%) underwent multilevel surgery based on the findings from DISE.
Ulualp et al. [34]	Retrospective chart review	82	Mean age of 6 ± 3.7 years	The study does not explicitly detail the patients’ previous surgical histories; however, it notes that the DISE was performed during the induction of anesthesia just before the patients underwent a tonsillectomy and adenoidectomy	Once an intravenous catheter was placed, sevoflurane was switched off and patients were given 1 mcg/kg of dexmedetomidine and 4 mcg/kg of glycopyrrolate while breathing 100% oxygen	VOTE classification	The study focuses solely on the intraoperative findings at the time of the DISE procedure and does not report on longitudinal postoperative follow-up	Original Research/Retrospective Study	The majority of children with OSA experienced airway obstruction at multiple sites. DISE is a valuable tool for identifying sites of upper airway obstruction that occur in addition to adenotonsillar hypertrophy.
Miller et al. [35]	Retrospective chart review	51	Mean 7.2 (±6.0 years SD)	Tonsillectomy-naïve (exclusion criteria: previous tonsillectomy or tonsils score 2+).	Inhalational sevoflurane and intravenous propofol per standard institutional protocol.	Chan–Parikh scale.	Pre- and post-operative PSG comparison	Original Clinical Research	DISE-directed surgery significantly improved mean AHI from 14.4 to 8.0 (*p* = 0.02) in children with small (1+) tonsils. Supraglottoplasty was the most common intervention (*n* = 23). Proved that clinically small tonsils do not typically cause obstruction, and DISE is crucial to avoid empirical AT.
Williamson [36]	Retrospective chart review	40	Mean age was 5.0 years, with a range of 8 months to 16 years	Any patients who had undergone prior surgical interventions for sleep apnea were excluded from the study	Propofol sedation was titrated and monitored by the anesthesia team, and spontaneous ventilation was maintained throughout the procedure in all cases	Internally developed, uniform template and scoring system	All patients were followed for a minimum of 3 weeks post-DISE or 3 weeks post-surgical intervention	Original Article	The study demonstrates that standard adenotonsillectomy is not always indicated for children with small tonsils, and DISE is a valuable tool to personalize surgical management and avoid unnecessary procedures
Mothersole et al. [37]	Retrospective chart review	27	Mean age of 7	Patients with a previous history of upper airway surgery were strictly excluded from the study	Pre-tonsillectomy and adenoidectomy (TA) drug-induced sleep endoscopy (DISE) was performed as patients emerged from sevoflurane while remaining deeply sedated with dexmedetomidine; anesthesia was maintained with a combined sevoflurane-opiate technique during the TA procedure	VOTE, ImageJ software was used to measure the percentage of airway obstruction on still images	The second DISE evaluation occurred immediately after the completion of the TA procedure	Original Research	Following TA, the percentage of obstruction at the level of the velum (decreased from 80% to 60%) and the oropharynx/lateral pharyngeal walls (decreased from 83% to 25%) was significantly reduced. The study concluded that most of the airway obstruction sites identified during pre-TA DISE persisted after TA in children with multiple sites of obstruction
Love et al. [38]	Retrospective chart review	79	Less than 2 years old	All patients were surgically naïve	The anesthetic technique utilized a combination of propofol and sevoflurane according to institutional protocols	Semi-validated Chan–Parikh scale	Post-DISE polysomnography follow-up	Original Research Article	After preprocedural DISE assessment, the majority (56.1%) of children with OSA and LM did not require standard adenotonsillectomy, yet they demonstrated similar improvements in their Apnea–Hypopnea Index (AHI)
Mendes et al. [39]	Retrospective study	80	Mean age was 4.3 years, with a standard deviation of 2.49	All 80 children underwent a primary surgery	Sedation was initially induced by inhaled sevoflurane and intravenous propofol was administered, followed by an infusion	Chan–Parikh scale	A repeat Type 1 polysomnography (PSG) was performed approximately 3 months (mean of 97.3 days) after the initial surgery.	Original Article	Obesity was significantly associated with surgical failure, making it the strongest predictor of persistent OSA after TA; targeted secondary surgery based on DISE findings was shown to be safe and highly effective.
De Lausnay et al. [40]	A single-center, retrospective study	70	0 to 18 years old. The mean age at the time of their first PSG was 3.5 years	Nine patients (12.8% of those with a lower airway anomaly and 15% of those without) had a history of previous upper airway surgery.	The specific pharmacological agents are not explicitly named in the text	Not mentioned	Follow-up PSGs were evaluated for 49 out of the 70 patients. The average time interval between baseline and follow-up PSGs was 10 months	Article	Lower airway anomalies were highly prevalent, documented in 70% (49/70) of the children (most frequently laryngomalacia, tracheomalacia, or combined malformations). There was no statistically significant difference in OSA prevalence, OSA severity, or treatment choices between children with lower airway anomalies and those with a normal airway endoscopy.
DeJarnette et al. [41]	A retrospective chart review	53	Average age was 8.8 years, with a range of 2 to 16 years	The study notes that 11 patients (20.8%) had a history of previous adenotonsillectomy.	The specific pharmacological agents used for sedation are not detailed in the provided text	VOTE classification	Patients underwent postoperative PSG and clinical follow-up.	Original Research Article	There was a statistically significant, though weakly positive, correlation between the intraoperative VOTE score and the postoperative Apnea–Hypopnea Index; no statistically significant differences in postoperative outcomes were found when comparing patients who underwent adenotonsillectomy versus other surgeries, or when comparing pharyngoplasty versus other surgeries.
Boudewyns et al. [42]	A retrospective analysis	28	Under 2 years old, with a median age of 1.5 years	Four children in the study group had a history of prior adenoidectomy	The specific pharmacological agents used for sedation are not detailed in the provided text	Obstruction of >50% at any given airway level was considered clinically significant. Specific structures were graded: adenoids, tonsils, hypotonia/circumferential collapse, anteroposterior epiglottis collapse, and late-onset laryngomalacia	The median time between the surgical intervention and the postoperative polysomnography (PSG) was 3.4 months	Retrospective clinical study	Adenotonsillar hypertrophy is the major cause of upper airway obstruction in this age group. DISE-directed upper airway surgery was curative (postoperative oAHI < 2/h) in 71.4% of the children.
Maksimoski et al. [43]	Retrospective chart review	24	The median age was 9.0 years, with an IQR of 4.0 to 14.0 years.	20 of the patients (83.3%) had a history of previous adenotonsillectomy	The text does not detail the specific anesthetic agents or sedation protocol used to induce sleep	Not mentioned	Patients were followed for at least 12 months after the procedure,	Original Research Article	The surgical intervention was successful in 84.6% of patients; the median relative reduction in oAHI following the procedure was 69.9%
Akkina et al. [44]	Retrospective chart review	24	Surgically naïve: mean 3.9 (±3.4 years). Prior AT group: mean 6.5 (±3.2 years)	10 had previous adenotonsillectomy (AT) (refractory OSA); 14 were surgically naïve.	Inhaled sevoflurane induction, followed by IV propofol maintenance; patient maintained spontaneous breathing.	Chan–Parikh scale	Pre- and post-operative PSG comparison (within 12 months)	Original Clinical Research	Confirmed a high proportion of multi-site upper airway obstruction in Down Syndrome patients with OSA. DISE-directed surgery led to overall improvements in PSG measures, with a statistically significant improvement in oxygen saturation nadir for children with previous AT (*p* = 0.04).
Chan et al. [45]	Retrospective case series	23	Mean 2.2 years (range 0.5–8.9 years)	No exclusion criteria; 70% of cohort had severe medical comorbidities (chromosomal anomalies, hypotonia, encephalopathy).	Transnasal flexible fiberoptic laryngoscopy under spontaneously ventilating propofol anesthesia in the supine position.	Chan–Parikh scale	Preoperative PSG compared with DISE scores; post-operative outcomes evaluated.	Instrument Validation and Clinical Research	First study to design and validate the Chan–Parikh scale. Showed substantial inter-rater and intra-rater reliability (70% of ICCs > 0.60). Higher total obstructive scores significantly correlated with lower oxygen saturation nadirs (*p* = 0.04) and multilevel obstruction (*p* = 0.02).
Lam et al. [46]	Retrospective case series	39	Mean 8.3 (±5.1 years SD)	Some had prior AT (refractory). Surgically naïve patients were included if they had small tonsils or high risk for residual OSA after AT.	Dexmedetomidine bolus (1–2 µg/kg) and infusion (0.5–1 µg/kg/h) combined with ketamine (1 mg/kg bolus, plus 0.25–0.5 mg/kg supplements).	Sleep Endoscopy Rating Scale (SERS)	Preoperative PSG compared with SERS total score. No postoperative clinical follow-up.	Original Clinical Research/Validation	SERS total score and sub-scores (oropharynx, hypopharynx) correlated significantly with baseline OSA severity (total score r = 0.75, *p* = 0.002). A SERS total score of ≥6 predicted severe OSA (OAHI > 10) with 81.8% sensitivity and 87.5% specificity.
Lam et al. [47]	Consensus development and validation study	25	Mean 8.6 ± 4.0 years (median 7.2)	Past surgical history: 29% had previous adenotonsillectomy, 6% palatoplasty/mandibular distraction, 65% none.	Multi-institutional standard protocols utilizing propofol or dexmedetomidine, maintaining spontaneous breathing.	International Pediatric Sleep Endoscopy Scale (IPSES)	Scale validation via inter-rater and intra-rater reliability across development and independent raters.	Collaborative Methodological Validation	IPSES consensus rating scale demonstrated moderate-to-excellent intra-rater reliability (best for lateral oropharynx, kappa 0.68–0.95) and moderate inter-rater reliability. Synthesizes previous scales (VOTE, Chan–Parikh, SERS) into a unified international scale endorsed by IPOG.
Mooney et al. [48]	Quality initiative (QI)	81	Not explicitly stated	Prior surgical data for the 81 subjects is not detailed	Premedication of intranasal dexmedetomidine, a quick “on-off” induction with sevoflurane/nitrous oxide for intravenous (IV) and flexible scope placement, followed by an IV bolus of dexmedetomidine prior to airway assessment	4-point Likert scale was utilized to evaluate the patient’s sedation level, tolerance of IV and scope insertion, and overall anesthesiologist and surgeon satisfaction	The study evaluates intraoperative variables up to 15 min of observation during the DISE procedure; long-term postoperative follow-up is not assessed.	Patient Safety/Quality Improvement (PS/QI) article	There was significant improvement across all measured aspects of the DISE procedure. Utilizing inhalational agents for IV access and flexible scope insertion in a rapid “on-off” fashion optimized patient tolerance regardless of the primary sedation medication. The final protocol yielded an overall median rating of 1 (excellent) for IV placement, scope insertion, and satisfaction from both anesthesiologists and surgeons (*p* < 0.01).
Kirkham et al. [49]	Single-center retrospective review	117	1 to <18 years old, with an average age of 6.5 years	Forty-one percent (41%) of the included children had a previous adenotonsillectomy (AT)	Anesthesia was maintained intravenously with either a continuous infusion of propofol or a dexmedetomidine load followed by continuous infusion. Ketamine was also co-administered variably in both groups	Chan–Parikh scale	Does not describe a postoperative longitudinal follow-up period	Original Article	The study found no statistically significant difference in the odds of experiencing ≥50% obstruction between the propofol and dexmedetomidine anesthetic groups at any level of the airway
Van de Perck [50]	A retrospective analysis of prospectively collected data	139	Median age 4.5 years	A total of 41 subjects (29.5%) had a history of prior upper airway surgery.	Propofol was administered via a bolus injection of 10 to 20 mg, followed by a continuous infusion of 6 to 10 mg/kg/h. In some older children, midazolam was used at the anesthesiologist’s discretion.	Obstruction of 50% or more at a given upper airway level was deemed clinically significant. The assessment specifically noted collapse at the adenoids, tonsils, tongue base, epiglottis, and palate.	Follow-up polysomnography (PSG) was performed three months after surgical interventions. For children receiving medical treatment, PSG was repeated after six months.	Original article	An increasing BMI z-score (higher body weight) is significantly correlated with circumferential upper airway collapse during DISE, independent of age and any history of upper airway surgery. The outcome of DISE-directed treatments was highly effective and similar across normal-weight, overweight, and obese children.

**Table 2:** Comparison of selected classification systems used in pediatric DISE.

Classification System	Anatomical Regions Assessed	Grading Scale	Limitations
VOTE [51]	Velum Oropharynx Tongue base Epiglottis	3-point (0–2)	Not included: nasal cavity adenoid larynx
Chan–Parikh [45]	Adenoid Velum Lateral walls/tonsils Tongue base Supraglottis	4-point (0–3)	Not included: nasal cavity Combines the epiglottis and other laryngeal structures into “supraglottis”
SERS [46]	Nose Adenoid Velum Lateral walls Hypopharynx Larynx	3-point (0–2)	Combines the tongue base and epiglottis into “hypopharynx”
NAVOTEL [25]	Nose Adenoid Velum Oropharynx Tongue Epiglottis Larynx	3-point (0–2)	None
PedDISE-8 [19]	Nose Adenoid Velum Tonsils Lingual tonsils Tongue base Epiglottis Aryepiglottic tissue	3-point (0–2)	None
IPSES [47]	Nose Adenoid Soft palate Lateral walls/tonsils Tongue base Epiglottis Arytenoid cartilages	3-point (0–2)	None

**Table 3:** Types of anesthesia used in pediatric DISE.

Anesthetic Agent	Role in DISE	Limitations
Propofol [6]	Primary intravenous agent: rapid onset/offset of action.	May exaggerate airway obstruction and does not replicate natural sleep.
Dexmedetomidine [6]	Primary intravenous agent; replicates NREM sleep.	Slow onset of action and risk of bradycardia/hypotension.
Ketamine [6]	Adjuvant, maintains muscle tone and reflexes.	Prolongs recovery time and increases salivation.
Sevoflurane [6]	Induction of anesthesia to establish IV access.	May exaggerate obstruction and must be discontinued quickly.

## Data Availability

No new data were created or analyzed in this study. Data sharing is not applicable to this article.

## Abbreviations

The following abbreviations are used in this manuscript:

AHI	Apnea–hypopnea index
AT	Adenotonsillectomy
BIS	Bispectral index
BMI	Body mass index
CPAP	Continuous positive airway pressure
DEX	Dexmedetomidine
DISE	Drug-induced sleep endoscopy
DISE-PAP	Drug-induced sleep endoscopy with positive airway pressure titration
DK	Dexmedetomidine and ketamine
IPSES	International Pediatric Sleep Endoscopy Scale
IV	Intravenous
MeSH	Medical Subject Headings
NAVOTEL	Nose, Adenoids, Velum, Oropharynx, Tongue, Epiglottis, Larynx
NREM	Non-rapid eye movement
OSA	Obstructive sleep apnea
PedDISE-8	Pediatric Drug-Induced Sleep Endoscopy grading system (8 sites)
PSG	Polysomnography
REM	Rapid eye movement
SERS	Sleep Endoscopy Rating Scale
VOTE	Velum, Oropharynx, Tongue base, Epiglottis
